# Large predatory coral trout species unlikely to meet increasing energetic demands in a warming ocean

**DOI:** 10.1038/srep13830

**Published:** 2015-09-08

**Authors:** J.L. Johansen, M.S. Pratchett, V. Messmer, D.J. Coker, A.J. Tobin, A.S. Hoey

**Affiliations:** 1 ARC Centre of Excellence for Coral Reef Studies, James Cook University, Townsville QLD 4811, Australia; 2 Whitney Laboratory for Marine Bioscience, University of Florida, St. Augustine, 32080, Florida, USA; 3 Red Sea Research Center, King Abdullah University of Science and Technology, Thuwal, Jeddah 23955, Kingdom of Saudi Arabia; 4 Centre for Sustainable Tropical Fisheries and Aquaculture, School of Earth and Environmental Sciences, James Cook University, Townsville, QLD 4811, Australia

## Abstract

Increased ocean temperature due to climate change is raising metabolic demands and energy requirements of marine ectotherms. If productivity of marine systems and fisheries are to persist, individual species must compensate for this demand through increasing energy acquisition or decreasing energy expenditure. Here we reveal that the most important coral reef fishery species in the Indo-west Pacific, the large predatory coral trout *Plectropomus leopardus* (Serranidae), can behaviourally adjust food intake to maintain body-condition under elevated temperatures, and acclimate over time to consume larger meals. However, these increased energetic demands are unlikely to be met by adequate production at lower trophic levels, as smaller prey species are often the first to decline in response to climate-induced loss of live coral and structural complexity. Consequently, ubiquitous increases in energy consumption due to climate change will increase top-down competition for a dwindling biomass of prey, potentially distorting entire food webs and associated fisheries.

More than a billion people worldwide are directly reliant on fisheries to satisfy their daily food requirements^1^,^2^ and demand for fisheries production will increase dramatically in the next two decades^3^. However, many tropical marine ecosystems are in rapid decline, owing in large part to the changing climate and increasing ocean temperatures^4^,^5^ with direct impacts on associated fisheries^3^,^6^. Importantly, most marine organisms, including fish and invertebrates, are ectothermic, and rising ocean temperatures will increase metabolic demand, and potentially push local conditions beyond thermal thresholds of individual species particularly in thermally sensitive systems such as tropical coral reefs^7^,^8^,^9^,^10^. To assess the capacity of ectothermic species, ecosystems, and food webs to persist in a warming ocean, the ability of species to increase energy intake and maintain productivity must be quantified across critical trophic levels.

To date, the majority of studies that have examined the effects of global warming on coral reef fish and fisheries species have focussed on the impact of habitat degradation, specifically the loss of live coral and associated structural complexity^6^,^11^,^12^. There is a relatively small, but growing body of work on direct physiological or behavioural demands on individual species^13^,^14^. Reef fish are ectotherms and all aspects of their metabolism and energy requirement is dictated by ambient temperature^15^,^16^,^17^. As temperatures increase, so does the rate of biochemical and cellular processes required for homeostatis, and the energetic cost of activity, growth and reproduction^17^,^18^,^19^. Increasing metabolic demands have to be balanced, either through increased food intake and/or reduced energy investment. Specifically, a species may capture larger or more energy rich prey to increase energy intake per meal, or consume more prey. However, these strategies will also require increased energy expenditure through greater hunting that may, in turn, increase vulnerability to predation and reduce individual fitness. Alternatively, a species may reduce activity and movement patterns to conserve energy^20^, effectively reducing its home range, the number of prey and predators encountered, and total energy intake. Regardless of the behavioural strategy, global warming is likely to alter the ratio of energy input to energy output with potentially deleterious effects for the health, growth and reproductive potential of individuals, species and entire functional groups.

Current understanding of how tropical coral reef fishes are likely to respond to increasing ocean temperatures is primarily based on studies of small, site-attached species^13^,^21^. These studies have all focussed on the ability of species to maintain physiological and functional performance or survive increasing temperatures. Consequently there is a lack of empirical evidence demonstrating if and how individual species, particularly larger predatory species, may overcome increased energetic needs (but see^22^). Although smaller individuals have higher mass-specific metabolic rates and energy requirements to maintain basal bodily functions and growth^23^, recent physiological studies have suggested that some of these short-lived coral reef species with rapid generation times may be able to acclimate to the rate of ocean warming^21^. In contrast, for larger and longer-lived species, which are assumed to have limited capacity to adapt over decadal timescales, it is likely to be their ability to behaviourally compensate for increased energetic needs that will determine susceptibility to increasing temperatures^24^,^25^. These larger bodied species are among the top predators on coral reefs^26^, are principal targets of coral reef fisheries, and an important source of food security and livelihoods on tropical coastal nations^3^. Any change to their foraging behaviour could directly impact multiple trophic layers of the food web, the health of the ecosystem, and the viability of reef-based fisheries.

Coral trout (*Plectropomus* spp) are among the most important coral reef fishery species, and also have an important ecological function as apex predators (trophic level >4)^27^. They are large (up to 125 cm total length, TL) piscivorous reef fishes, which are heavily targeted throughout their range due to their high commercial value^28^,^29^,^30^. In the Indo West-Pacific, the common coral trout (*P. leopardus*) is the major finfish fisheries target constituting more than 22% of all coral reef finfish catches on Australia’s Great Barrier Reef (GBR)^31^. Consequently, the degree to which ocean warming may impact the health, growth and reproduction of this and other valuable species, and the sustainability of their fisheries, will likely depend on the ability to change foraging patterns to meet increasing energy demands.

To examine the capacity of an apex predator to acclimate and adjust feeding behaviour to increasing ocean temperatures and increasing energetic needs, we conditioned a mix of 112 wild caught individuals of common coral trout (*P. leopardus*) from northern (warm-water) and southern (cold-water) populations on the GBR to one of four different temperatures (24, 27, 30 or 33 °C). These temperatures were chosen based on current summer and winter averages experienced by each population and a +3 °C increase *sensu* the Intergovernmental Panel on Climate Change’s emissions stabilization scenario RCP 4.5 by 2100^5^. We also assessed whole ecosystem impacts of increasing energetic needs under global warming by comparing measured changes in feeding behaviour with predicted changes in prey availability on tropical coral reefs.

## Results

**Figure 1:**
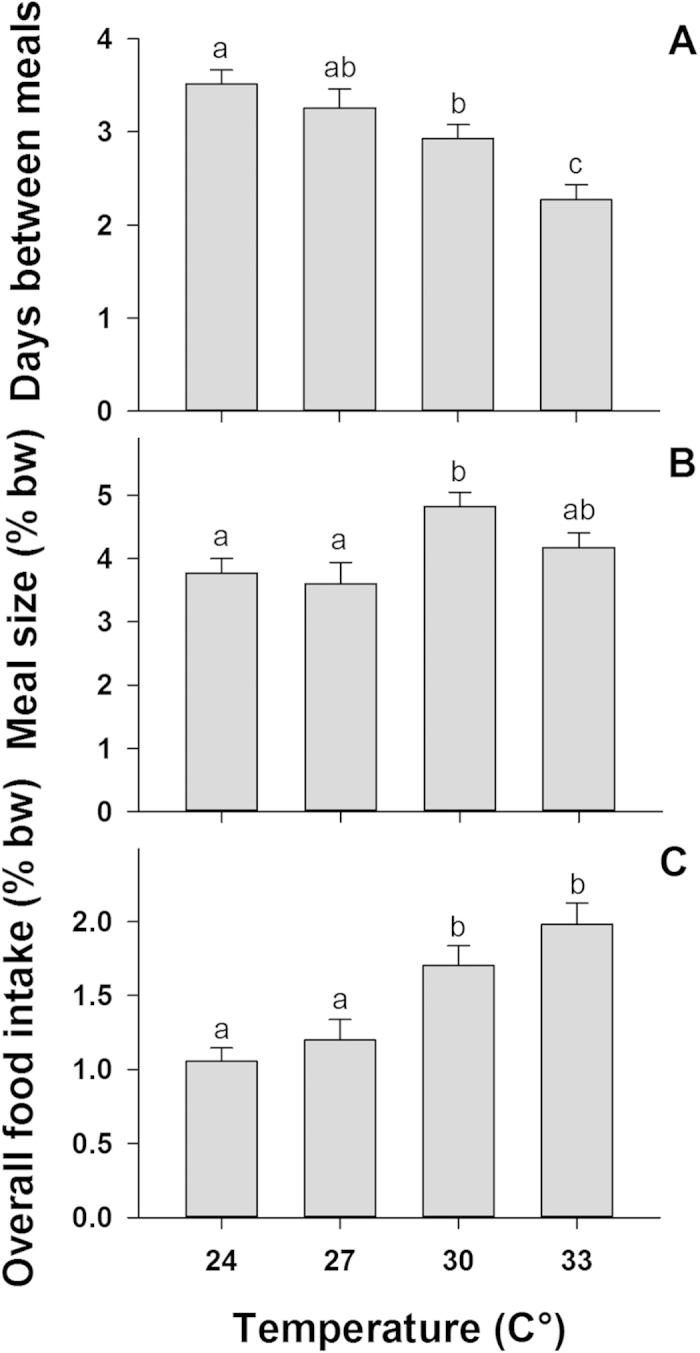
The feeding frequency, meal size and average overall food intake of common coral trout (*Plectropomus leopardus*) across four temperature treatments . Values of meal size and overall food intake are in % body-weight (% bw) and error bars are standard error of the mean. Significant differences across temperatures are indicated with letters above each column. (**A)** shows the number of days between meals; (**B)** shows average meal size, and (**C)** shows the average overall food intake at each temperature treatment.

Following six weeks of slow acclimation and conditioning, before experiments began, we quantified food intake of individually tagged fish fed to satiation every 1–2 days over a 21-day period, recording the feeding frequency (i.e. days between meals), meal size (i.e. total mass of all food pieces consumed at each feeding session relative to body weight which was measured at the beginning and at the end of the trial) and overall food intake (i.e. cumulative weight of all food pieces consumed/feeding frequency). We found that temperature had a significant positive effect on feeding frequency (F_3,108_ = 10.61, p < 0.001), meal size (F_3,108_ = 6.72, p = 0.001), and overall food intake (F_3,108_ = 14.93, p < 0.001, Fig. 1). Every 3 °C increase in temperature led to a 1.15-fold increase in the overall feeding frequency, from feeding on average once every 3.5 ± 0.2 days at 24 °C to once every 2.3 ± 0.2 days (mean ± SE) at 33 °C, and a 1.23-fold increase in average overall food intake from 1.1 ± 0.1% body-weight per day (%bw/day) at 24 °C to 2.0 ± 0.2%bw/day at 33 °C. Meal size showed a minimal change from 3.8 ± 0.2%bw at 24 °C to 4.2 ± 0.2%bw at 33 °C. While coral trout gained 6.5 ± 1.8% body-weight at 27 °C (F_2,108_ = 5.69, p = 0.001) over the experimental period, there was no significant increase or reduction in the body-weight of trout at the other temperature treatments (supplement S1), showing that food intake was sufficient to maintain body-weight across all temperatures.

**Figure 2:**
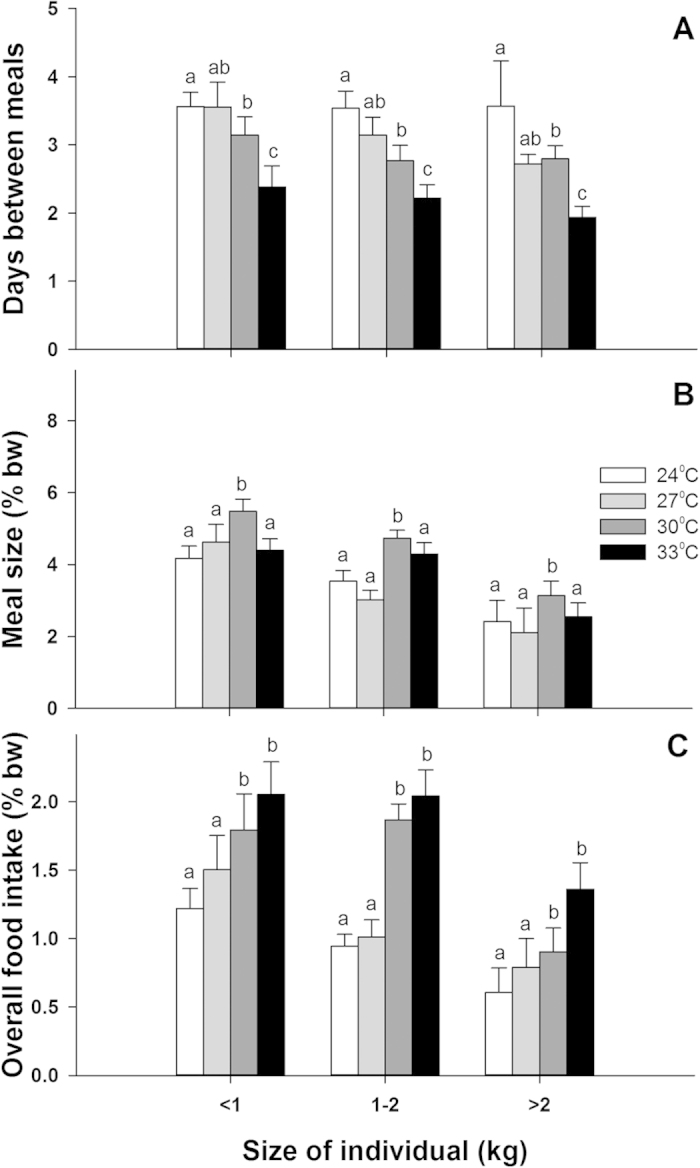
The effect of body size on (A) the feeding frequency, (B) meals size and (C) average overall food intake of common coral trout (*Plectropomus leopardus*) across four temperature treatments. Feeding frequency is in days, while meal size and overall food intake are in % body-weight (% bw). Error bars are standard errors of the mean. Significant differences within size groups and across temperatures are shown above each column. Column shadings (white to black) represent different temperatures. Notice how temperature affects all size-groups equally.

Body size of fishes had a significant negative effect on meal size (F_2,108_ = 22.23, p < 0.001) and overall food intake (F_2,108_ = 7.98, p < 0.001, Fig. 2, Supplementary Table S1). Relative to body size, small (<1 kg) and medium (1–2 kg) individuals consumed more than large individuals (>2 kg), averaging 4.6 ± 0.2%bw/meal (equating to 1.6 ± 0.1%bw/day) in small individuals and 2.5 ± 0.3%bw/meal (0.9 ± 0.1%bw/day, mean ± SE) in large individuals. From 24 °C to 33 °C, small individuals increased food intake from 1.2 ± 0.1 to 2.1 ± 0.2%bw/day, while large individuals increased from 0.6 ± 0.2 to 1.4 ± 0.2%bw/day (mean ± SE, Fig. 2). This equated to an average increase in food intake of 1.19–1.34 times for every 3 °C temperature rise. There was no significant interaction between body size and temperature (F_6,108_ = 0.99, p = 0.436, Fig. 2), showing that the responses of fishes to increasing temperature were consistent across all size classes.

**Figure 3:**
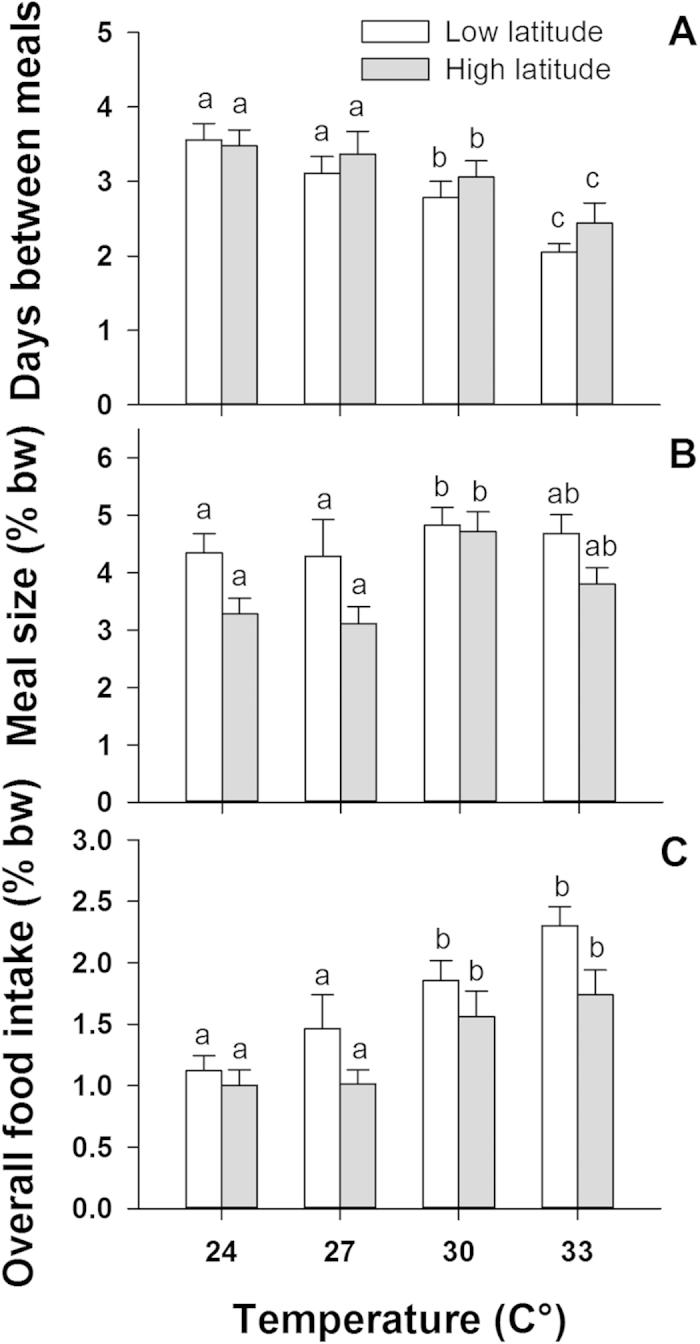
Differences in (A) feeding frequency, (B) meal size and (C) average overall food intake between a low latitude (warm water) and a high latitude (cold water) population of common coral trout (*Plectropomus leopardus*) across four temperature treatments. Values of meal size and overall food intake are in % body-weight (% bw) and error bars are standard error of the mean. Significant differences within and across temperatures and populations are shown above each column.

Low (14 °S) and high latitude (23 °S) populations showed no significant difference in body-size (F_3,108_ = 0.72, p = 0.584), the frequency of feeding (F_1,108_ = 0.79, p = 0.375), or weight change (F_1,108_ = 1.20, p = 0.277) between temperature conditions. In spite of these similarities, the low latitude (warm-water) population consistently ate larger meals and consumed more food per day across all temperatures examined (Meal size: F_1,108_ = 14.92, p < 0.001; Overall food intake: F_1,108_ = 8.82, p = 0.004, Fig. 3). On average, the low latitude population ate 4.5 ± 0.2%bw/meal and 1.6 ± 0.1%bw/day, relative to 3.7 ± 0.2%bw/meal and 1.3 ± 0.1%bw/day by the high latitude population (mean ± SE, Fig. 3). This indicates that populations may be able to adjust to 3 °C increases in temperature, allowing the warm-water population to consume 22% more food in every feeding event, without eating more frequently than the cold-water population.

## Discussion

This is the first study to empirically demonstrate that tropical reef fish can regulate energy intake to compensate for increasing thermal metabolic requirements at elevated temperatures^16^,^32^, at least when food is plentiful. Empirical studies have shown that a 10 °C increase in temperature causes a 2–3 fold increase in the rate of biochemical enzyme-catalysed reactions and whole-organism metabolism in marine ectothermic fishes^19^ which equates to a predicted 23–39% increase in energetic demand for a 3 °C temperature rise. These rising costs of metabolic processes must be met with appropriate increases in food intake to maintain long-term health, growth, and reproduction at elevated temperatures (see e.g., 33). Our results demonstrates that a 3 °C increase in ocean temperatures, expected in the tropical Pacific by 2100^5^, will require significant increases in the frequency of feeding and average overall food intake for large predatory and commercially important coral reef fish, such as the common coral trout (*Plectropomus leopardus*). Although the impact may vary between species^33^, the 1.23-fold increase in overall food intake recorded here is consistent with an expected 1.2–1.4 fold increase in energy need associated with a 3 °C temperature rise^19^. These findings demonstrate that food availability will be a fundamental determinant in the responses of large predatory fishes to ocean warming.

Increased food intake at higher temperatures may help some populations compensate for higher energy requirements for metabolic maintenance, growth and reproduction. However, the acquisition of energy underpins species survival and ecology of all reef ectotherms, and coral reef ecosystems may be unable to sustain such ubiquitous increases in predation rates. Considering elevated temperatures just 2–3 °C above the long term average have been shown to negatively affect many reef organisms^7^,^34^, and lead to climate induced habitat degradation through loss of live coral and structural complexity of reef habitats, the abundance of many of the smaller prey fishes and invertebrates are at risk of declining by more than 50% with ocean warming^4^,^6^,^11^,^12^,^35^. Specifically, it is estimated that over 19% of the world’s coral reefs are already lost, and 54% of reefs may be lost within the next 20–40 years^36^, particularly at equatorial locations where summer temperatures are already approaching the thermal maximum of many organisms including fish and coral^34^,^37^. Empirical studies of species richness and abundance following coral bleaching events or habitat degradation have repeatedly shown large scale reductions of 60–75% across the majority of reef fish families, including coral trout and other fished species that are not directly dependent on coral for food or shelter^11^,^12^,^35^,^38^,^39^. Consequently, it is improbable for all remaining trophic levels in the ecosystem to uniformly ramp up productivity by the required 23–39%. More likely, such drastic changes in food availability will increase top-down competition for a dwindling number of prey, reducing the density and carrying capacity for higher trophic levels and lead to distorted food webs as well as emigration of species to cooler-deeper habitats or higher latitudes^40^.

Interestingly, each population of the common coral trout predominantly attained its increased food intake by increasing the frequency of feeding events, rather than increasing meal sizes. This may indicate that individuals already eat to satiation or maximum digestive capacity whenever possible befitting the feast and famine lifestyle of many predatory fishes^41^. Alternatively, increased foraging frequency may be a direct response to increased digestion rates at higher temperatures^42^. Irrespective of the cause, increased foraging frequency is likely the most viable means of increasing energy intake in the short-term, although this will also increase energy requirements for pursuing additional mobile prey beyond that recorded here. Interestingly, this study also found slight differences in meal size between cold and warm water populations, suggesting a plasticity to increase meal size over longer timescales, and potentially also the size and range of prey species, which may ultimately help some populations of coral trout maintain health and survival under global warming.

The observed changes in feeding rate may also have far reaching implications for the sustainability of fisheries on large predatory species such as coral trout. Juvenile and small individuals have higher mass-specific energy requirements to maintain bodily functions and growth^23^ meaning that there will be even greater energetic effects on these life history stages. Reduced foraging and greater energy expenditure at elevated temperature may reduce size-at-age, while reduced abundance of prey will lead to fewer large individuals available for fisheries^43^,^44^. Indeed, fisheries of coral trout on the Great Barrier Reef are significantly more productive at higher (>16 °S) latitudes^45^ where ocean temperatures remain around 27 °C during summer, coinciding with higher growth rates. Although widely distributed in the Indo-Pacific, the growth rates, stock density and catch per unit effort (by number and biomass) are reduced at warmer locations^45^,^46^, suggesting that the effects of elevated temperature on coral trout fitness and productivity are already evident across latitudes. Importantly, increased demand for feeding and reduced feeding opportunities under ocean warming may also make individuals more likely to take a bait and therefore more susceptible to fishers^47^. As such, ocean warming may bring short-term gains in catches, which will need to be carefully managed to avoid over-exploitation and collapse of already threatened stocks.

We are only beginning to understand the possible ramification of global warming on aquatic food webs. Given the vital role apex predators play in ecosystem health and fisheries worldwide, it is critically important that we gain a comprehensive understanding of factors that may threaten these species. This study is the first to demonstrate the plasticity of a model tropical fished species, the common coral trout, to regulate energy consumption according to ambient temperature. This plasticity and behavioural regulation of food intake bodes well for the long-term survival of individuals and populations. However, the impact of increasing water temperature on ontogenetic growth, reproduction and abundance, as well as the wider impacts of the increased feeding on the ecosystem and the fisheries they support, are relatively unexplored. If the increase in food requirement demonstrated here is mirrored in other predatory fishes, such escalations of predation pressures will undoubtable have cascading effects through the entire ecosystem.

## Methods

This work was approved by the Animal Ethics Committee (AEC) at James Cook University and carried out in accordance with James Cook Animal Ethics Approval No. A1723. Collections were carried out under Great Barrier Reef Marine Park Authority collection permit G10/33239.1 and Department of Primary Industries Fisheries Permit 103256.

### Study species and location

112 adult common coral trout, *Plectropomus leopardus* (Family Serranidae), ranging in size 38.2–69.5 cm TL, were used for this study. In total, 59 fish (47.6 ± 9 cm, 1497 ± 950 g, mean ± SD) were caught in the vicinity of Heron Island and the Swains (23 °S) in the southern Great Barrier Reef (GBR), and 53 fish (45.5 ± 5 cm, 1109 ± 430, mean ± SD) were caught north of Cooktown, GBR (14 °S). Mean water temperature where these species routinely live at <9 m depth differ by ca. 3 °C year round, averaging 23.9 °C in winter and 29.3 °C in summer in the northern GBR, compared to 20.8 °C and 27.0 °C in the southern GBR. All fish were transported to the James Cook University Marine and Aquaculture Research Facilities Unit (MARFU) in Townsville, Qld, Australia, within 72 h of capture.

### Experimental protocol

Upon arrival at MARFU, all fish were marked with two multi coloured T-bar anchor tags (Hallprint, Hindmarsh Valley, SA, Australia) in a unique combination of colour and position to visually distinguish individuals and populations. Individuals were then randomly distributed across eight 2000 L round tanks (120 × 150 cm, height × diameter) at a stocking density of maximum 20 fish per tank. This density is less than 50% of that used by aquaculture and the live fish trade to keep coral trout healthy in captivity. All tanks were held under natural 13–11 h light–dark regime (subjected to sunrise and sunset as beginning and end of daylight) and continuously supplied with filtered seawater (salinity 34 ppt) at an initial temperature of 24 °C. After 4–5 days, each tank was allocated to one of four temperature treatments (24, 27, 30, and 33 °C) with two tanks per temperature. The temperature in each of treatment tanks was increased at a constant rate of 0.5 °C/day until reaching the desired temperature (±0.2 °C SE). All fish were held at experimental temperatures in fully oxygenated water for six weeks before experiments began to ensure full metabolic and behavioural acclimation. The length and weights of all individuals were measured at the beginning of the acclimation period and again after 10 weeks.

Throughout the slow acclimation period and subsequent 21 day experimental period, individuals were fed to satiation every 1–2 days, with ca. 24 g pieces of *Nemipterus* spp. and ca. 10 g pieces of *Acanthochromis polyacanthus*. All individuals from all treatments were fed on the same days. During every feeding session, single pieces of prey were dropped into the experimental tank and the amount and type of food eaten by each individual was visually recorded based on their unique T-bar tags. To account for competition between individuals, food was provided to individuals and added to the tank until a total of 3 pieces of prey remained uneaten for 15 min, indicating that all individuals were satiated.

### Statistical analysis

To standardize for size-differences between individuals, meal size and overall food intake were calculated as a proportion of body-weight (e.g. food eaten (g)/body-weight (g)). We calculated mass-specific growth rates of each individual based on log differences in initial and final weight (i.e. (ln mass2 − ln mass1)/(t2 − t1)). Initial size differences between populations and temperature treatments were examined using a two-way ANOVA with temperature and population as fixed factors. Differences in feeding frequency, meal size, average overall intake and growth rates were then compared using a main-effects 2^nd^ degree factorial Nested General Linear Mixed model. Tanks were nested within temperature, temperature and population were treated as fixed factors, and body size (<1 kg, 1–2 kg, >2 kg) was treated as an ordered covariate. We selected the most parsimonious models by using maximum likelihood estimation, removing non-significant variables one by one if their removal did not result in a significantly larger Akaike information criterion (AIC). Significance of main effects were estimated using Markov Chain Monte Carlo (MCMC)^48^. MCMC is robust to the fact that the exact degrees of freedom cannot be calculated in linear mixed-model designs^48^, but can be estimated based on treatment groups (df1 = k − 1) and samples (here reported as df2 = n − 1). To assess the validity of the generated models, we performed likelihood ratio tests comparing the models with fixed effects to null models with only the random effects. Assumption of homoscedasticity and normality were examined using analysis of residuals, and log transformations were used where necessary. Specific differences within and among temperatures, populations and size categories were examined using post hoc pairwise comparisons of covariate-adjusted means. False detection rate was used to correct for Type I errors^49^. Individuals that refused to eat were excluded from analysis. All data were analyzed using the R packages *lme4*^48^, *LMERConvenienceFunctions*^50^ and *lsmeans*^51^ (R Development Core Team 3.1.2, 2014).

## Additional Information

**How to cite this article**: Johansen, J.L. *et al.* Large predatory coral trout species unlikely to meet increasing energetic demands in a warming ocean. *Sci. Rep.* **5**, 13830; doi: 10.1038/srep13830 (2015).

## Supplementary Material

Supplementary Information
