# Supplementary material for: Large predatory coral trout species unlikely to meet increasing energetic demands in a warming ocean
Source: Sci Rep. 2015 Sep 8;5:13830. doi: 10.1038/srep13830 (PMC4561880; doi:10.1038/srep13830)
Supplement: Supplementary Information [file srep13830-s1.pdf]

Supplement S1

Title:

Large predatory fisheries species unlikely to meet increasing energetic demands in a warming ocean

Authors:

J.L. Johansen*[1,2], M.S. Pratchett[1], V. Messmer[1], D.J. Coker[1,3], A.J. Tobin[4], A.S. Hoey[1]

Table S1: Relative weight change (%)

Temperature (°C)	24		27		30		33	
Range	min	max	min	max	min	max	min	max
Low latitude	-30.0	5.8	-2.9	21.8	-17.6	24.2	-8.1	11.7
High latitude	-7.3	10.8	-17.1	19.9	-6.6	10.9	-12.8	13.6

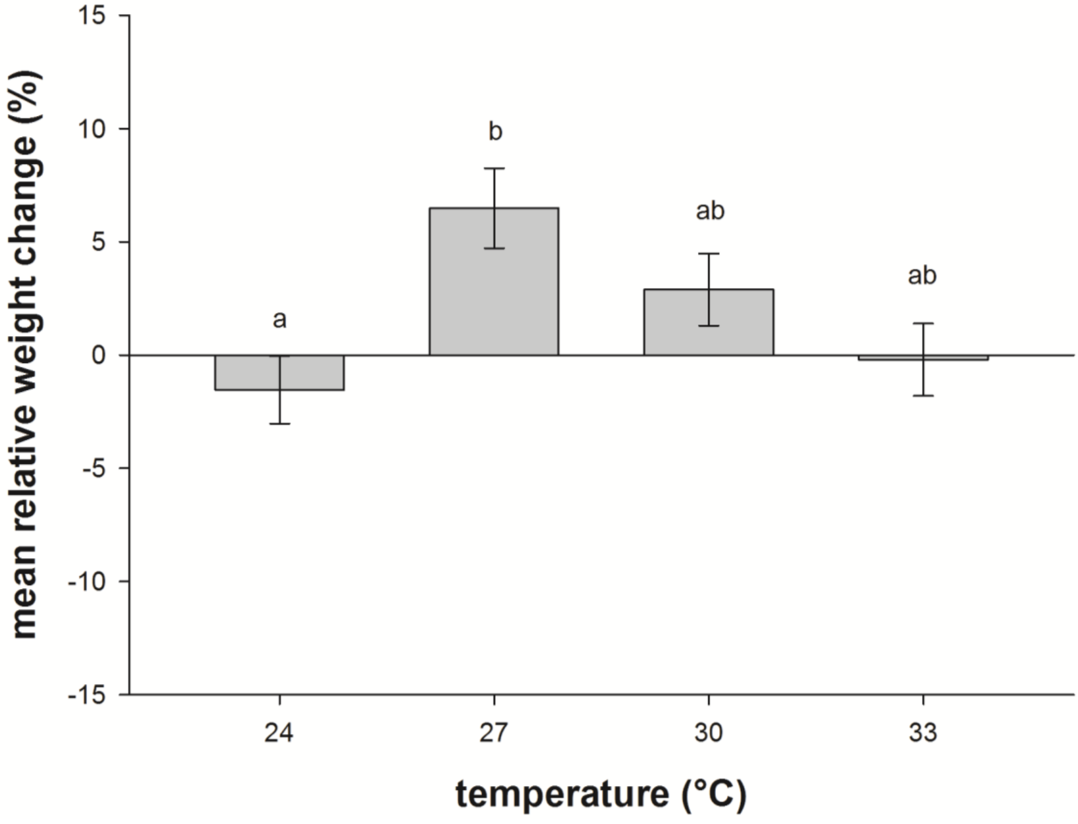

Figure S1. The mean relative weight change of coral trout (*Plectropomus leopardus*) after 21 days of ad-libitum feeding at four different temperatures (27-33°C). Error bars are SE and significant differences between temperatures are marked above each column.
